# The interaction of fluorescent nanodiamond probes with cellular media

**DOI:** 10.1007/s00604-017-2086-6

**Published:** 2017-01-27

**Authors:** Simon R. Hemelaar, Andreas Nagl, François Bigot, Melissa M. Rodríguez-García, Marcel P. de Vries, Mayeul Chipaux, Romana Schirhagl

**Affiliations:** Department of Biomedical Engineering Antonius Deusinglaan 1, University Medical Center Groningen, Groningen University, 9713 AW Groningen, Netherlands

**Keywords:** Fluorescent nanodiamonds, Aggregation, Surface analysis, Cellular uptake, Corona formation, Imaging, Microscopy, Nanoscale sensing, Proteins

## Abstract

Fluorescent nanodiamonds (FNDs) are promising tools to image cells, bioanalytes and physical quantities such as temperature, pressure, and electric or magnetic fields with nanometer resolution. To exploit their potential for intracellular applications, the FNDs have to be brought into contact with cell culture media. The interactions between the medium and the diamonds crucially influence sensitivity as well as the ability to enter cells. The authors demonstrate that certain proteins and salts spontaneously adhere to the FNDs and may cause aggregation. This is a first investigation on the fundamental questions on how (a) FNDs interact with the medium, and (b) which proteins and salts are being attracted. A differentiation between strongly binding and weakly binding proteins is made. Not all proteins participate in the formation of FND aggregates. Surprisingly, some main components in the medium seem to play no role in aggregation. Simple strategies to prevent aggregation are discussed. These include adding the proteins, which are naturally present in the cell culture to the diamonds first and then inserting them in the full medium.

**Electronic supplementary material:**

The online version of this article (doi:10.1007/s00604-017-2086-6) contains supplementary material, which is available to authorized users.

**Graphical abstract:**
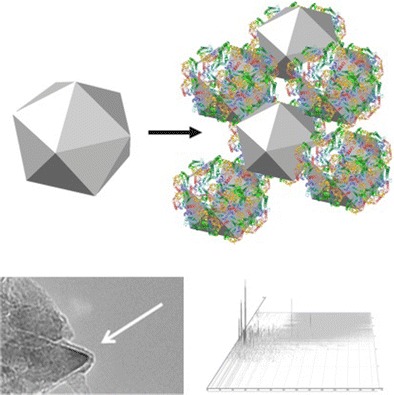
Schematic of the interaction of nanodiamonds with cell culture medium. Certain proteins and salts adhere to the diamond surface and lead to aggregation or to formation of a protein corona.

## Introduction

The nitrogen vacancy center (NV-center), a lattice defect in diamond, is responsible for the stable, non-bleaching fluorescence of the diamond. It acts as a versatile quantum sensor [1]. Fluorescent nanodiamonds (FNDs) have already been used to measure several quantities including magnetic resonances, [2] temperature, [3, 4] pressure and to detect external NMR and ESR signals as well as electric or magnetic fields [5, 6]. Depending on the diamond surface quality, magnetic fields, for instance from a single electron spin, [7] can be detected up to tens of nm from the defect. First attempts for nanoscale intracellular measurements have been made for temperature [8] and magnetic resonances [9, 10]. As a sensor probe, FNDs have several advantages: they allow unprecedented spatial resolution, [3, 7] they are non-toxic [11] while they also have a modifiable surface [10, 12]. The field of using nanoparticles for bioimaging has been reviewed by Wolfbeis [13].

However, when introduced to cellular media, formation of aggregates occurs, thus greatly increasing the hydrodynamic diameter of the particles. This phenomenon is particularly relevant as aggregates reduce cellular uptake, since endocytosis is size dependent [14]. Furthermore, it is desired for sensing applications to have the FND probe as close as possible to the analyte of interest, which is impeded by the aggregation process. Forming of aggregates or the formation of a protein layer on the diamond can increase the distance between the NV center and the target molecule. It has to be noted that our study is different from well-known aggregation in detonation nanodiamonds [15, 16]. These aggregate during synthesis without the presence of other molecules. We did not investigate detonation nanodiamonds in our study.

**Fig. 1:**
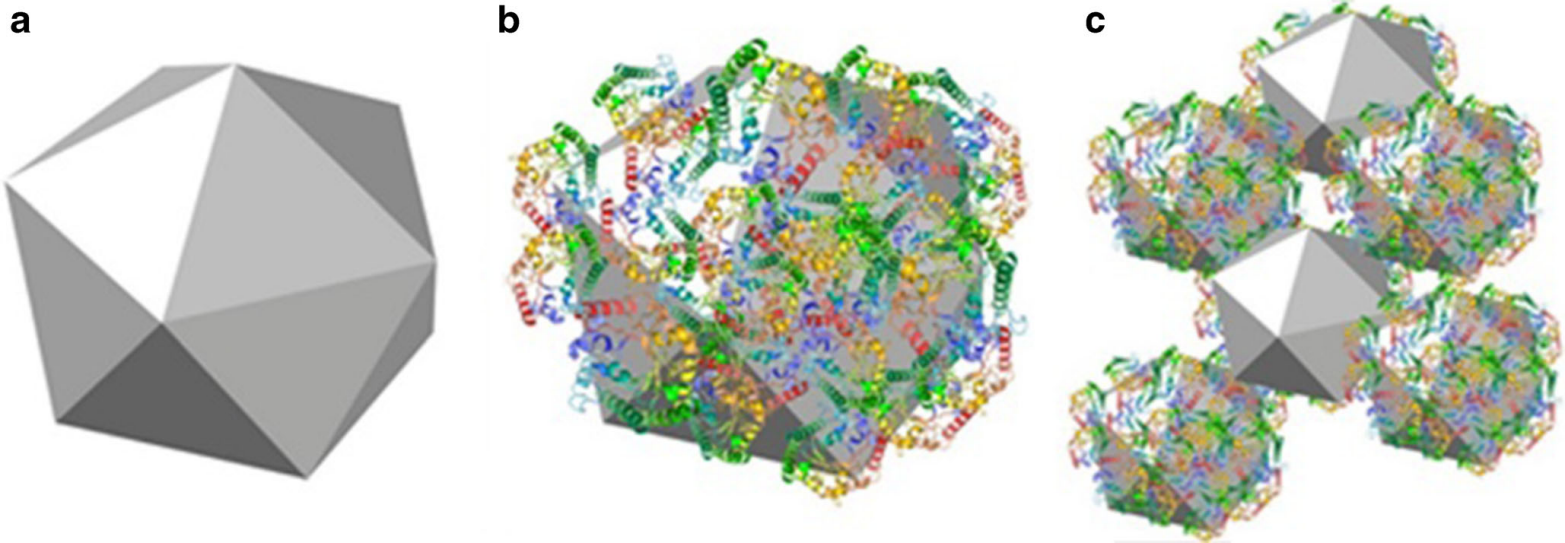
Schematic representation of nanodiamond aggregation. **a** depicts the bare Nanodiamond particle. In figure **b** the nanodiamond particle with proteins adhering to the surface is shown. Figure **c** represents the nanodiamond aggregation by interaction between multiple diamond particles through protein connections

For NDs, until now only salting out was considered in physiological salt conditions [17]. In cellular media, however, the situation is much more complex. For various nanoparticles, the formation of a so-called “protein corona” of medium components is known [18–21]. It determines the nanoparticle’s physicochemical properties, including hydrodynamic size, surface charge, and aggregation behavior [22]. Proteins have already been used as coating for diamond particles [23–26]. Furthermore, the adhesion of proteins to diamond has been utilized in protein separation [27, 28]. However, which of the naturally present proteins in cell medium adhere to the diamond surface plays a role in intracellular sensing applications has not been studied. Here we first observe and characterize protein corona formation and the aggregation phenomenon for diamond nanoparticles. The complex interplay between the medium components leads to the formation of aggregates of notable size (see Fig. 1).

We investigated the influence of salts and proteins present in the medium in the aggregation process and suggest strategies to avoid or mitigate the aggregation phenomenon. We analyzed size distributions, protein presence and the surface composition of the aggregates. While working with severely aggregated particles is completely impossible for most medical applications, for the typical applications of FNDs (as sensing different quantities in cell biology) some aggregation is tolerable [29]. Thus we not only looked at dispersed particles but considered it important to also analyze the composition of aggregates. Some ground breaking work has been done without considering aggregation or corona formation, [30] however, for future applications it is important to account for this effect when interpreting signals or when overcoming the current limits of the technique.

## Materials and methods

### Fluorescent nanodiamond (FND) starting material

FNDs with a diameter of 25 nm (later abbreviated as FND_25_) purchased from Microdiamant AG (Lengwil, Switzerland, MSY 0–0.05 μm GAF, reference: 129,578, www.microdiamant.com), are acid cleaned and have an oxygen terminated surface. These diamond particles have a zeta potential of approximately −22 mV (see also **Table S1**). The surface charge is very important for the interaction of the diamonds to other molecules [31, 32]. These are particles with a flake structure [33] which were used as received for all experiments except for cell uptake because they are the most commonly used material for magnetometry applications. They are currently the smallest diamonds available that can be engineered so that every diamond hosts an NV center. For cell uptake we used FNDs (end concentration 1 μg mL^−1^) with a diameter of 70 nm (later abbreviated as FND_70_) for better visibility due to the high amount of NV centers. They were purchased from Adamas Nanotechnologies, Inc. (NC, USA; ND-NV-70, >300 NV centers / diamond particle, www.adamasnano.com) and used as received.

### Aggregate/corona formation

We chose to analyze the interaction with DMEM (Dulbecco’s Modified Eagle Medium) since it is the most common standard cell medium for culturing mammalian cells. It is a complex mixture providing cells with nutrients. It contains amino acids, vitamins, salts, antibiotics as well as bovine serum (which itself contains a complex mixture of over 3700 proteins). Aggregates were created by dispersing the FNDs_25_ to an end concentration of 200 μg mL^−1^ in different media. The different media were (1) DMEM Complete (consisting of DMEM + complements: Glutamax (1%), Pen/Strep (1%), Foetal bovine serum (FBS) (10%), Gibco Life Technologies, Bleiswijk, the Netherlands, www.thermofisher.com/ch/en/home/brands/gibco). (2) DMEM without complements and (3) pure or diluted (10%) FBS. To wash samples, we centrifuged for 10 min at 12.000 xG. Then we discarded the pellet and added distilled water, shaked it and sonicate it for 10 mins, resuspende the pellet. The centrifugation was repeated and the pellet was removed once again. The final aggregates where either resuspended again or dried to perform further experiments.

### Characterization of size and appearance

#### Dynamic light scattering

Dynamic light scattering (DLS) measurements were performed using a Malvern ZetaSizer Nano system (Malvern Instruments Ltd., Malvern, UK, www.malvern.com) to determine the hydrodynamic diameter of the particles. Samples were measured at least in triplicate in folded capillary cells and mixed intermediary to prevent sedimentation of larger particles. Sizes were calculated using the average number mean. We are aware of the fact that DLS is not ideal to determine sizes of large heterogeneous aggregate particles. Nevertheless, we found the technique very useful to screen whether or not aggregation took place qualitatively and too some extend quantitatively. The size of larger particles we also confirmed by TEM.

#### Electron microscopy

Bare FNDs_25_ were prepared on a silicon surface and visualized using SEM (pictures taken in a Leo 1530 Gemini, Carl Zeiss AG, Jena, Germany, www.zeiss.com). This was done by diluting the stock solution 1:200 in methanol and dropping 5 uL of of this solution onto a 1 × 1 cm silicon wafer piece. Inlens detection and a voltage of 10 kV were used to record the images. FNDs_25_ aggregates with DMEM complete medium were prepared on a holey carbon coated grid (Quantifoil 1.2/1.3, Quantifoil, Jena, Germany, www.quantifoil.com). This was achieved by placing the grids for 5 min on a drop of the solution, removing the grid and drying for a view minutes in air. Finally, we imaged with a TEM Philips CM12 (Philips, Eindhoven, The Netherlands, www.philips.com) equipped with a slow CCD camera to show how the proteins assemble around the crystalline diamonds (the identity of the FNDs was confirmed by selected area electron diffraction (SAED)).

### Characterization of composition of particles

#### Mass spectrometry

The following samples have been investigated: (1) FBS (as control sample), (2) 10% FBS + FNDs (3) DMEM + FBS + FND. Samples (2) and (3) were also analyzed after washing. Samples were prepared and then washed in DI water or using a sucrose cushion. This latter method [27] removes the ‘soft’ protein corona. Afterwards all samples were freeze dried and prepared for HPLC and MS/MS. For a detailed description of the technique, please review **text S1** (supporting information).

#### X-ray photoelectron spectroscopy

Three samples were prepared by mixing diamond and medium: (a) Pure FND, (b) FND in DMEM, (c) FND in DMEM +10% FBS, freeze dried and analyzed using a S-Probe (Surface Science Instr., Mountain View, CA, USA). For a more detailed methodology, please review **text S2** (supporting information).

### Characterization of behavior during uptake

HeLa cells (grown in DMEM complete medium) were incubated with 1 μg mL^−1^ FNDs_70_ for 5 h at 37 °C, 5% CO2. The FNDs_70_ were prepared by dispersing them in DMEM or DMEM complete. Alternatively a sample was prepared by first mixing the diamonds in 100% FBS (end concentration 10%) followed by resuspension in DMEM. Afterwards cells were fixed with 3.7% PFA and permeabilized using 1% Triton X-100 in PBS (containing 0.9% NaCl,). Cells were stained using Phalloidin-FITC (stains the actin cytoskeleton) and DAPI (stains the nucleus). Cells were imaged using a Zeiss LSM780 microscope (Zeiss, Jena, Germany, www.zeiss.com). Standard settings were used for imaging the respective dyes. Since diamond particles do not bleach, they were imaged after imaging the dyes using the highest gain settings. Diamond particles are excited with a 532 nm laser and emit a broad band above 600 nm. Magnification steps were made to visualize diamond particles.

## Results and discussions

### Size distribution

**Fig. 2:**
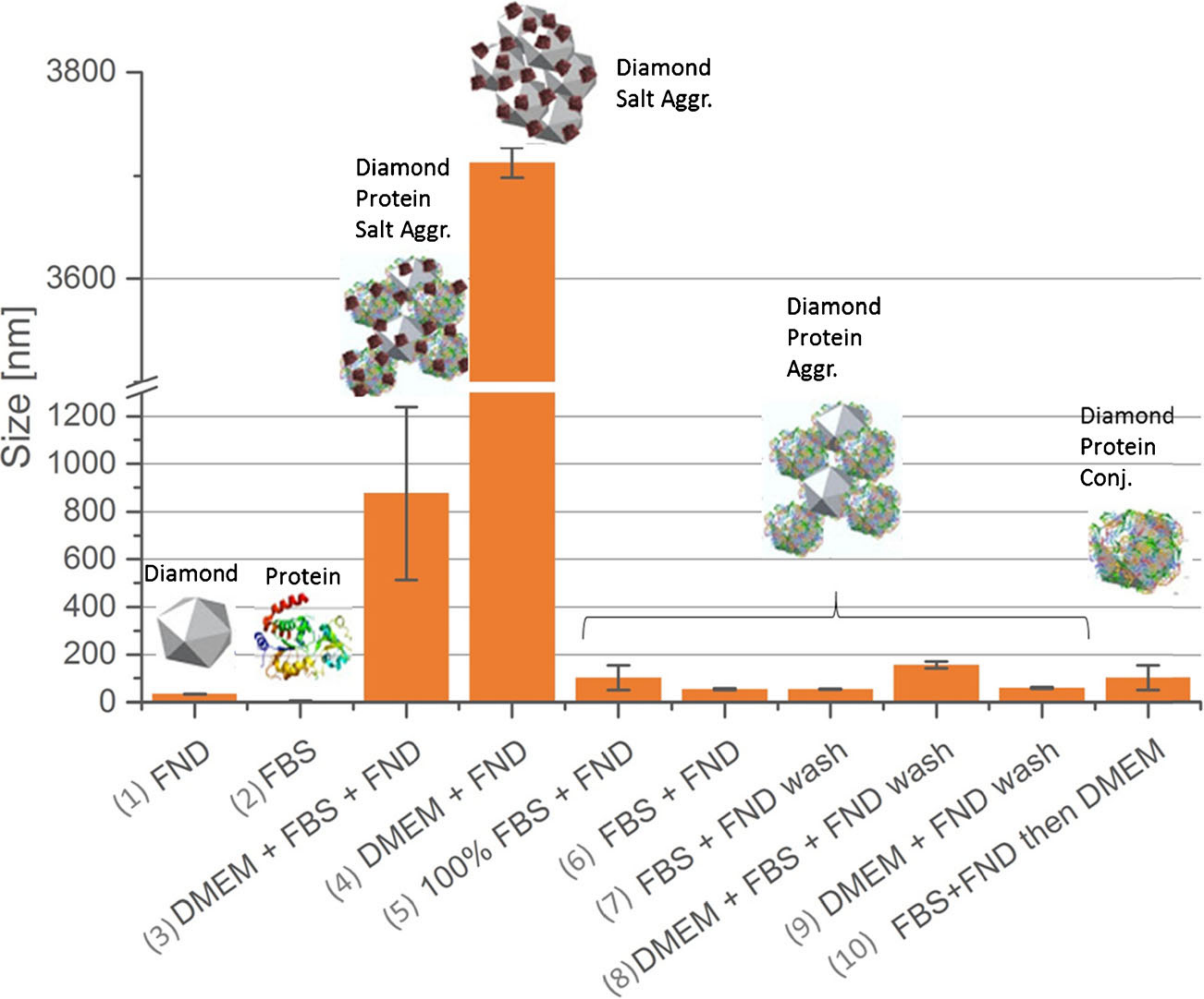
Hydrodynamic diameters measured using cumulant analysis for ND particles (Microdiamant MSY 0–0,05, hydrodynamic diameter 25 nm) in different media. Error bars correspond to the standard deviation. The schematics on top show the composition of the particles we found. Samples (1) and (2) are reference measurements, the diamonds were suspended in water. If not stated differently the concentration of FBS was 10% (prepared in distilled water to eliminate the salt effect). The samples which are labeled with “wash” are measured after resuspending the particles. For the exact measurement protocol see the supporting information

The first parameter we analyzed was size and appearance of our particles. This was first done by DLS measurements. Figure 2 shows the results of these measurements.

To investigate which part of the sample is involved in aggregation we tested the components FNDs_25_ in water (1) and FBS (2) as reference, and the complete DMEM medium (3) as well as composites formed from diamonds with different parts from the medium: the uncomplemented DMEM medium (4), 100% FBS (5) and 10% FBS (6). Thus, influence of salts as well as proteins on the hydrodynamic diameter was explored. To investigate how stable these aggregates are and to find out if washing is useful to reduce aggregate sizes we also washed the samples with DI water. The size data ((7)–(9)) in Fig. 2 were taken after redispersing the washed particles in water. In the last row in Fig. 2 we first mixed the FNDs_25_ with FBS and then added the particles with the protein corona to DMEM medium. This method turned out to be useful to prevent aggregation.

The measurements in DI water yielded particle sizes close to the supplier information and no big increase was observed. Thus we conclude that the starting material (in agreement with the claims of the supplier) does not aggregate in water. However, when adding different medium components the sizes increase due to either formation of a corona or aggregation. Aggregates formed from adding diamonds to salt solution (4) showed the highest hydrodynamic diameter (in the micron regime) compared to aggregates containing diamonds and proteins (samples (5) and (6)) or aggregates containing diamonds, proteins and salts (sample (3) and (10)). In sample (3), which represents the conditions in which the cells are normally cultured, we find relevant aggregation, which is however, still lower than in sample (4). Indeed proteins mitigate the aggregation tendency to a certain degree. Introducing FNDs first to proteins to allow the formation of the protein corona followed by adding the DMEM (sample (10)) is efficiently reducing the aggregation. Washing reduces the aggregate sizes (most efficiently in sample (4). The corresponding zeta potential as well as polydispersity indices of measured particles can be found in Table S1.

### Aggregate morphology

**Fig. 3:**
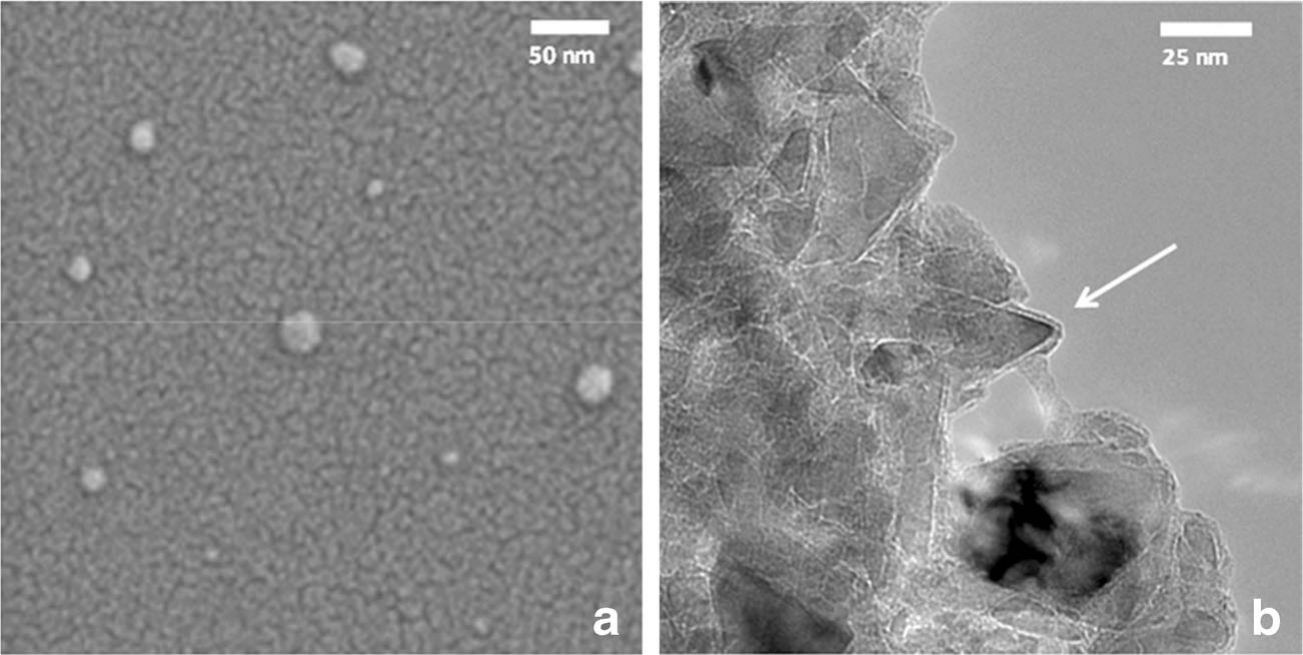
Electron microscopy images of FND and FND aggregates. Fluorescent nanodiamonds spotted on a silicon surface and imaged with an SEM (Leo 1530 Gemini, Carl Zeiss AG) (3**a**). FND aggregate imaged using Philips CM12 (Philips, Eindhoven, The Netherlands) on holey carbon grids (3**b**). The arrow indicates the protein corona

Imaging of the aggregates using electron microscopy revealed huge aggregates with proteins between the FNDs_25_. In Fig. 3a dispersed FNDs_25_ (as received) can be seen on a silicon surface under a SEM (sample (1) from Fig. 2). The samples were imaged at least 3 times and different areas were chosen for imaging to avoid imaging artefacts in order to find representative areas for imaging. Figure 3b shows the proteins assembling around the crystalline diamonds (on a holey carbon coated grid), imaged with a TEM Philips CM12 (sample (3) from Fig. 2).

### Composition of aggregates.

The presence of proteins on the aggregates was confirmed by Fourier transformed infrared spectroscopy (in attenuated total reflection mode and matrix-assisted laser desorption/ionization, see Fig. S4). Proteins were analyzed after trypsin digestion using a label free mass spectrometry technique (for a detailed description of the analysis methods see text S1 and Fig. S2) with a semi-quantitative assessment of relative protein amounts using normalized spectral counts [34–36].

A large number of different proteins were found in the aggregates. Interestingly, we find a great range of proteins that participate in the aggregation process that are not among the most abundant proteins of the serum, thus suggesting a certain selectivity of the nanodiamond surface towards some proteins. This selective adsorption of certain proteins onto nanoparticles is also commonly observed for other nanoparticles [19]. While in pure FBS only a total number of 25 proteins were identified. The reason is that the sample is dominated by the most abundant proteins (also known from literature [34, 37]) to be serum albumin and Alpha-2-HS-glycoprotein, which make up 66% of the normalized spectral counts. Relatively rare proteins, however, can be found in the aggregates.

**Fig. 4:**
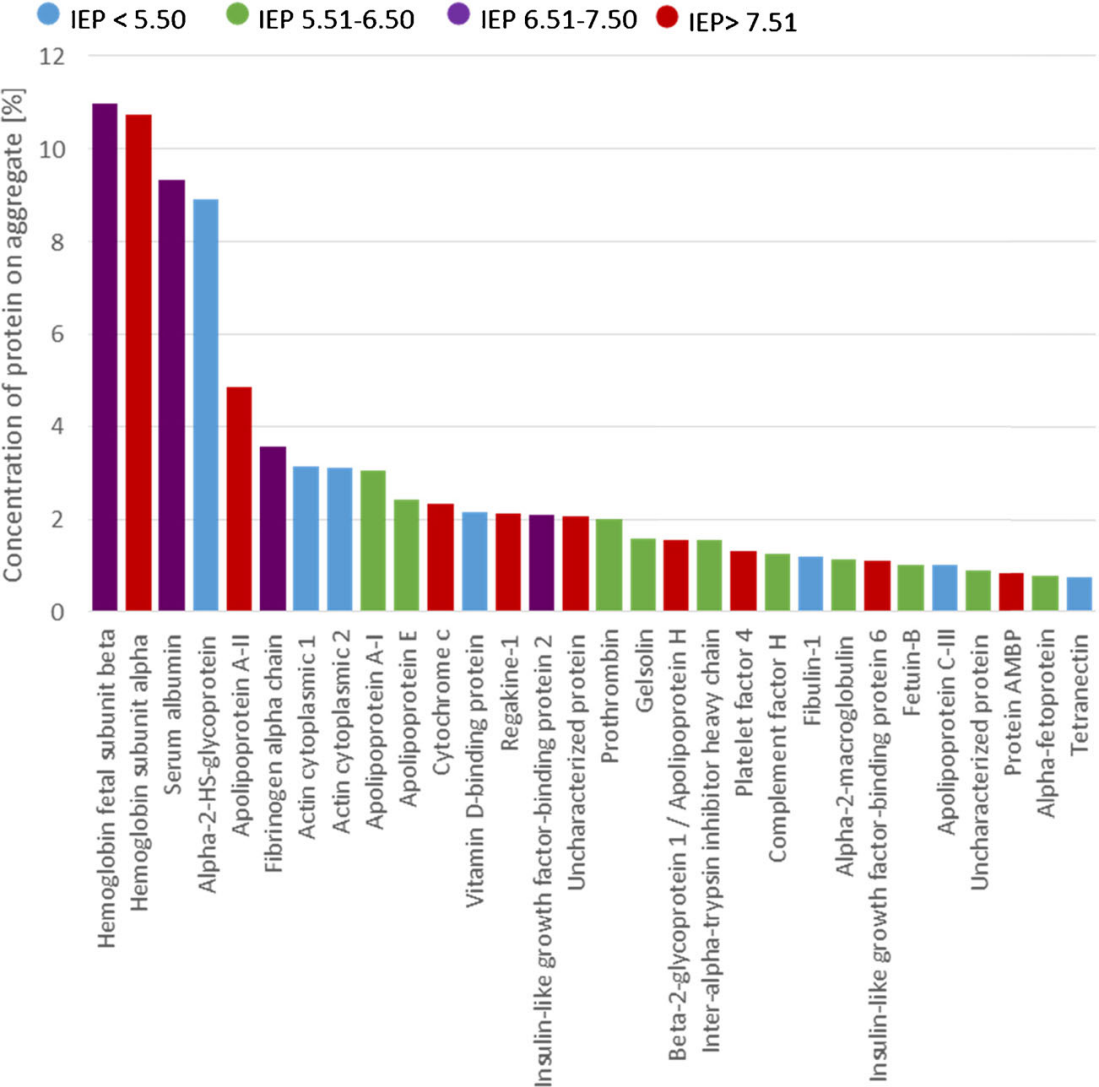
Most abundant proteins and their theoretical isoelectrical point present in aggregates from FND in DMEM +10% FBS (sample 3) calculated from a typical mass spectrum (for details see supplementary information)

Figure 4 shows an overview of the most abundant proteins we found in the medium aggregates. For a detailed overview of the most important proteins in the medium and on the respective aggregate, their properties and their functions, see table S2. In sample DMEM + FBS + FNDs 66 proteins were identified. Similar numbers of proteins involved in the corona formation have also been found for other nanoparticles [38]. This reflects the complexity of the components involved in the aggregation process (and the crucial role of inorganic salts). Washing of samples with DI water decreases the amount of proteins by washing away the “soft” corona (loosely bound proteins). Only proteins with higher affinity remain on the diamond surface. While before washing the sample is still dominated by more abundant proteins, after washing we can identify more of the low abundance proteins. This results in over 200 proteins, which were identified. We investigated these proteins further to find any similarities between binding or non-binding proteins. Surprisingly, no correlation between the adsorption and the theoretical isoelectric point (IEP) (see Fig. 4), (from http://web.expasy.org/compute_pi/) or the molecular mass of the proteins was established. Also, no significant difference between the IEP of the 25 most abundant proteins in the medium and in the diamond aggregates was found. This is in agreement with similar studies on other nanoparticles [39, 40]. It can be explained by the fact that proteins have an inhomogeneous distribution of charges at their surfaces. Even with an overall negative net charge of the protein, positive charge domains may allow an electrostatic interaction with the particle surface. For multiple layers protein-protein interactions also have to be taken into account, possibly reducing the importance of the charge and polarity of the nanodiamonds. Proteins present in the aggregates, which are marked green in Table S2 show a molecular function related to binding to negative compounds (e.g. heparin or ATP). This is a possible explanation for the favored adsorption on the oxygen-terminated FNDs with a negative zeta potential. An overview of the proteins found in the aggregates is given in Fig. 4.

Additionally, we investigated proteins that are even tighter bonded to the diamond particles. These were separated by using a cleaning method suggested by Docter et al. [38] To this end the aggregates were centrifuged through a sucrose cushion to completely remove loosely bound proteins. The results (which were qualitatively similar to Fig. 4) as well as some more details on the method are shown in Fig. S3.

### Surface analysis of bare particles

The analysis of bare nanodiamonds reveals oxygen groups on the surface of the diamond (hydrogen is not shown in XPS analysis). This conclusion is also supported by the FTIR spectra of the bare particles. Although diamond bands dominate the spectrum also some surface groups are visible. The broad band at 3600 cm^−1^ most likely comes from O-H groups. C = O is visible as a shoulder of the diamond peak at 1700 cm^−1^. The peak at 1100 cm^−1^ indicates the presence of C-O groups. This indicates the presence COOH and other oxygen containing groups, which corresponds to the manufacturers carboxylation of the particle.

### Aggregate surface analysis

**Fig. 5:**
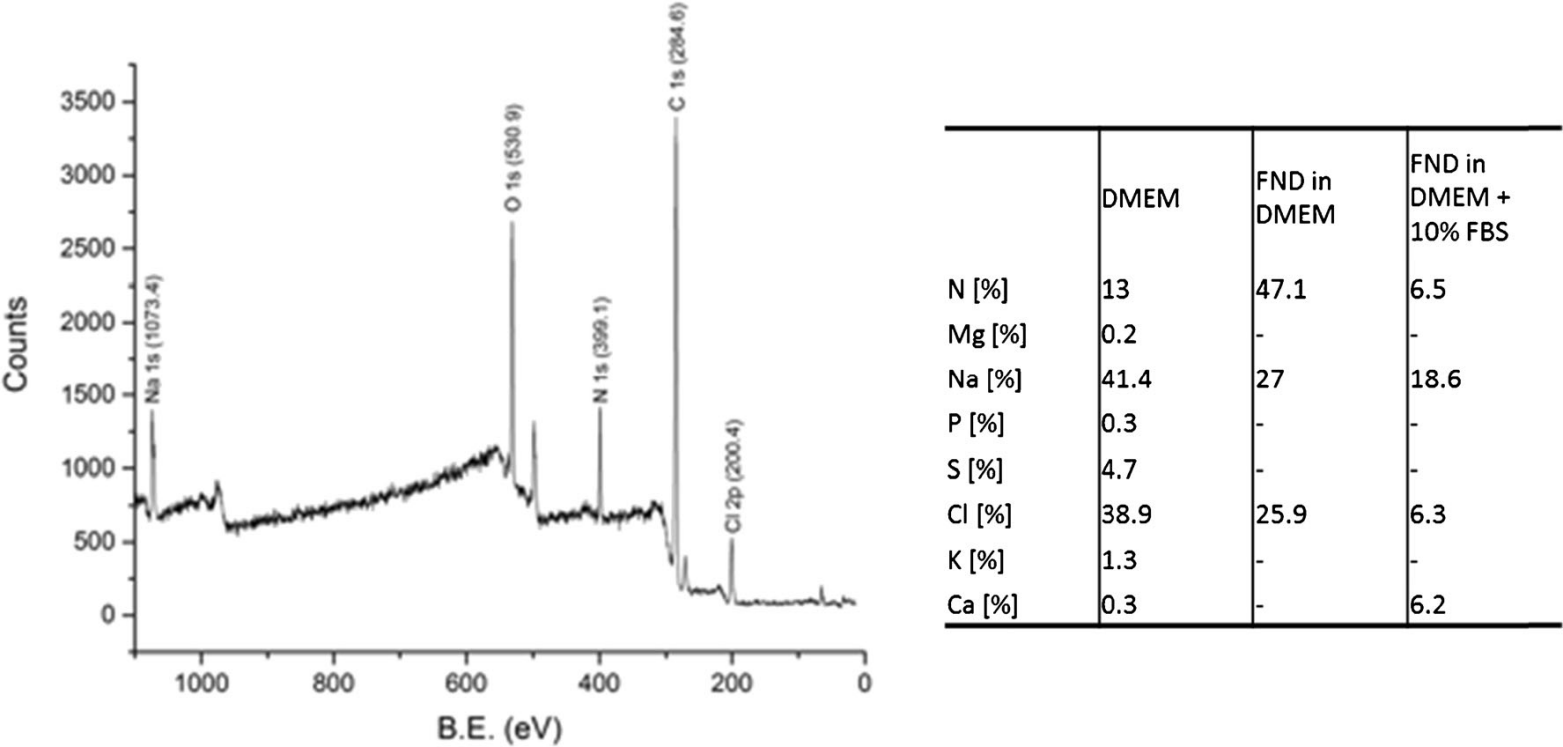
Determining the salt contribution in aggregates: left: XPS spectrum of FND in DMEM +10% FBS (sample 3). Also here, sodium chloride remains the main compound in the aggregates (apart from the carbon mainly present as diamond, protein and amino acids). Right: Comparison of the element composition of DMEM medium, and the measured elemental ratios in aggregate samples

The aggregates were analyzed using X-Ray Photoelectron Spectroscopy in order to determine the element composition (especially inorganic salts involved). For the results see Fig. S3 and table S2. Sodium chloride is the major salt component in the aggregates. While this is not surprising, calcium, magnesium, potassium and phosphorus – although in great quantity present in the medium - seem to be less abundant (if at all) in the aggregates. Together with the fact that far less aggregation is observed when leaving out sodium chloride this suggests a central role of sodium chloride in the aggregation process. Figure 5 lists the elements presents in the measured samples compared to the salts in DMEM.

### Prevention of aggregation

A simple method we found to improve aggregation is adding the FBS to diamonds first. Once a thin coating of proteins has formed on the diamond surface they can safely be introduced in the full medium. Coating the diamond with proteins from FBS is an effective option to prevent inter-particle aggregation and achieve size reduction. Indeed, the resuspension of FNDs_25_ in FBS and following dilution in DMEM (end concentration 200 ng mL^−1^) showed an average size 90 nm, resembling the situation of diamonds in water or in 10% FBS. Compared to other methods to prevent aggregation of nanodiamonds coating with FBS first and then adding the coated protein into DMEM has an advantage; no additional proteins are introduced. If additional proteins are introduced, an exchange of proteins can occur when inserted in the final medium.

### Cellular uptake

**Fig. 6:**
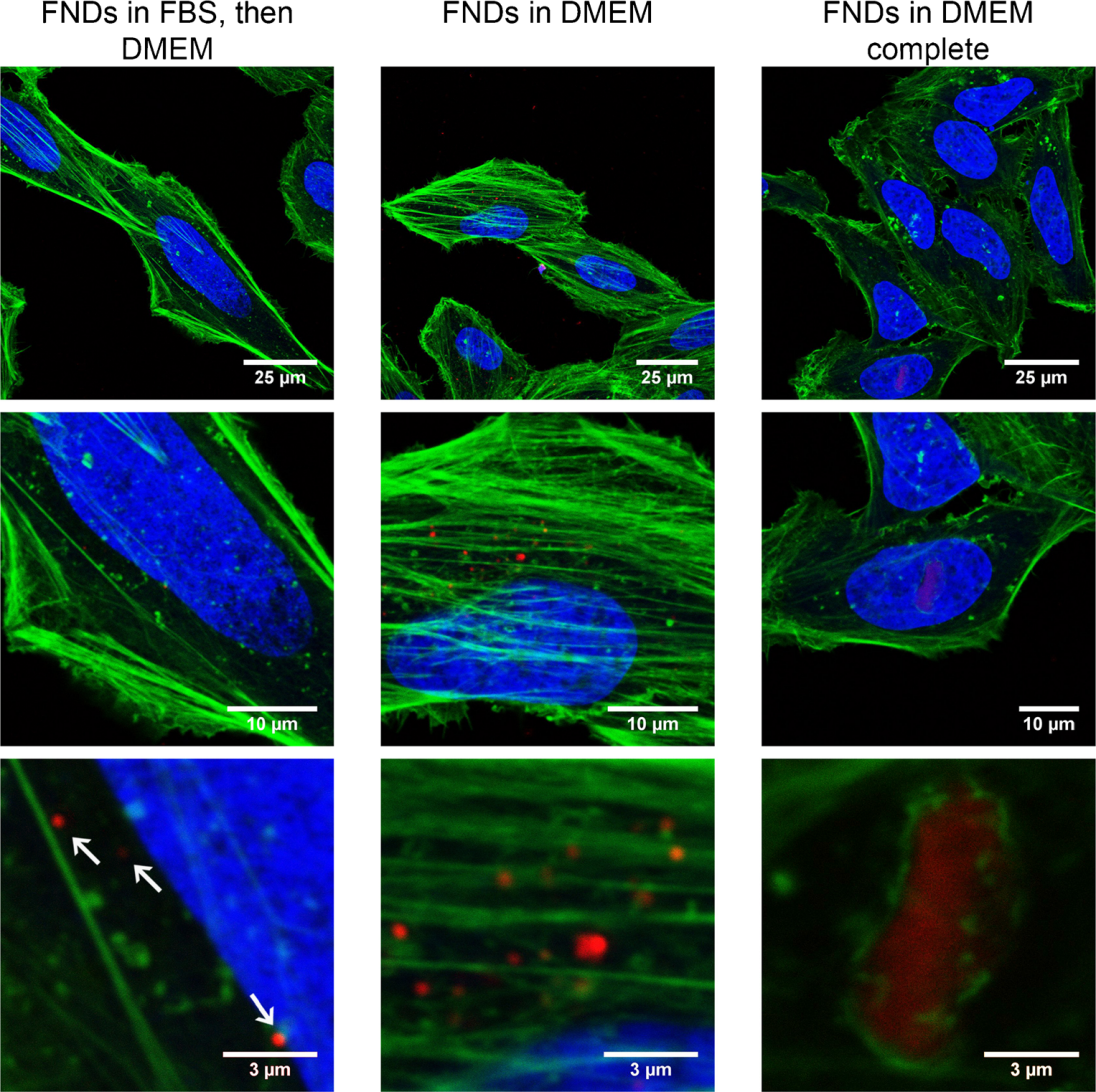
Cellular uptake of nanodiamonds into HeLa Cells. (*red*: nanodiamonds, *green*: Phalloidin-FITC (stains the actin cytoskeleton), *blue*: DAPI (stains the nucleus)). Arrows indicate diamond particles. In the lower right pane, a large diamond aggregate (occupying almost the entire area of the pane) can be seen precipitating on the cell (as it is surrounded by the actin filaments of the cytoskeleton) (DAPI was omitted to make the diamond more visible)

The reduction of fluorescent nanodiamond aggregate size greatly increases chances of having a single FND taken up inside a cell. We tested the impact of aggregation on uptake into HeLa cells using FNDs with a 70 nm diameter (manufacturer information, on average). The results of these uptake experiments are shown in the confocal image Fig. 6, with the cells actin cytoskeleton in green, the nucleus in blue and the diamonds in red.

In the serum-free DMEM medium, there are more aggregates. In the DMEM complete medium hardly any diamond particles are taken up, however a large aggregate is shown which is precipitating on the cell (there is part of the cellular membrane around it, lower right pane, DAPI signal not shown for visualization purposes). These images are typical for the whole sample, indicating that uptake of the aggregates in HeLa cells is possible. However, for other cell types and sensing applications, single diamonds are preferred. With the resuspension of FNDs_70_ in FBS before DMEM, we were able to obtain the smallest (diffraction limited) particle signal (indicating no/very little aggregation). Other options to achieve size reduction might encompass lower particle concentrations or using an inert solution as medium. Alternatively, using a less concentrated medium or a medium lacking NaCl and certain proteins found in the aggregates might prevent or improve aggregation as well.

## Conclusions

Here we have for the first time performed an in depth analysis of the composition of FND aggregates and protein coronas for use in biological systems. The consideration of different sizes of diamond particles is very important in achieving the most efficient uptake in cellular systems. In this research we have mainly analyzed 25nm particles, since these approach the limit of diamond size with stable NV centers inside. Although larger particles will have a lower surface to volume ratio, we believe that the aggregation is mostly determined by the surface chemistry. These results can thus be extrapolated to larger particles with the same surface composition. We provide a detailed analysis of which components contribute to the aggregation and which components form a corona on the diamond surface. This information can be taken into account in the future when sensitivity is estimated. Furthermore, we suggest a simple method to improve aggregation. When particles are first suspended in Foetal Bovine Serum and then diluted in DMEM, the resulting particle size decreases. In this situation the interactions between different protein-diamond aggregates is prevented through coating the diamond with FBS, before adding to a salt rich solution. The LC-MS/MS data shows that different proteins adhere to the diamond surface and thus participate in the aggregate formation. Ultimately, we have shown that the presence of salts in a protein rich environment results in much larger aggregates (compared to an environment without salts). 

## Electronic supplementary material

ESM 1 (DOCX 451 kb)
