# Supplementary material for: The interaction of fluorescent nanodiamond probes with cellular media
Source: Mikrochim Acta. 2017 Jan 27;184(4):1001–9. doi: 10.1007/s00604-017-2086-6 (PMC5346409; doi:10.1007/s00604-017-2086-6)
Supplement: Supplementary file 1 — (DOCX 451 kb) [file 604_2017_2086_MOESM1_ESM.docx]

**Electronic Supplementary Material**

**The interaction of fluorescent nanodiamond probes with cellular media**

Simon R. Hemelaar, Andreas Nagl, François. Bigot, Melissa M. Rodríguez-García, Marcel P. de Vries, Mayeul Chipaux, Romana Schirhagl*

*University Medical Center Groningen, Groningen University, Department of Biomedical Engineering Antonius Deusinglaan 1, 9713 AW Groningen, Netherlands*

* E-mail: [romana.schirhagl@gmail.com](mailto:romana.schirhagl@gmail.com)

**Table S1.** Average Zeta-potential in mV and standard deviation for each sample are displayed. Zeta potential of all samples reduced significantly after washing (P < 0.01), Furthermore the polydispersity indices are listed.

| Sample | Mean | Std. Deviation | PdI |
| --- | --- | --- | --- |
| 25nm FND | -22,20 | 3,439 | 0.19 ± 0,07 |
| FBS | -15,95 | 0,520 | 0.53 ± 0.06 |
| FBS + FND | -18,13 | 0,208 | 0.18 ± 0.00 |
| FBS + FND washed | -27,90 | 1,153 | 0.31 ± 0.02 |
| FBS + DMEM + FND | -9,85 | 0,882 | 0.52 ± 0.06 |
| FBS + DMEM + FND washed | -28,08 | 0,591 | 0.52 ± 0.06 |
| DMEM + FND | -19,83 | 1,193 | 0.26 ± 0.07 |
| DMEM + FND washed | -37,43 | 0,306 | 0.12 ± 0.00 |


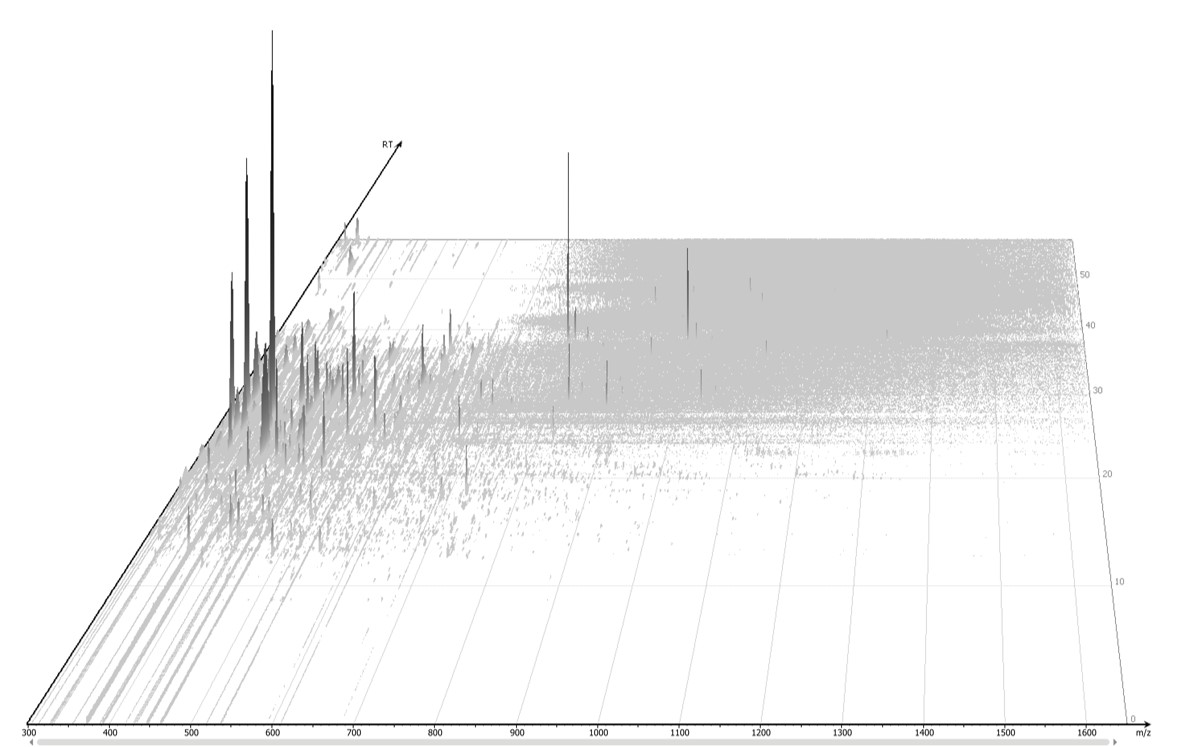


**Figure S1. Example of a chromatogram of the protein corona of nanodiamond particles after trypsin digestion.** The X-axis shows the M/Z while the Y-axis shows the retention time of the column. The height of the peaks corresponds to the intensity of the signal in MS/MS

**LC-MS/MS methods**

Samples were analyzed by nanoLC–MS/MS on an Ultimate 3000 system (Dionex, Amsterdam, The Netherlands) interfaced on-line with a Q-ExactivePlus mass spectrometer (ThermoFisher Scientific., San Jose, CA). Peptide mixtures were loaded onto a 5 mm × 300 μm i.d. trapping micro column packed with C18 PepMAP100 5 μm particles (Dionex) in 2% AcN in 0.1% FA at the flow rate of 20 μL/min. After loading and washing for 3 minutes, peptides were back-flush eluted onto a 15 cm × 75 μm i.d. nanocolumn, packed with C18 PepMAP100 1.8 μm particles (Dionex). The following mobile phase gradient was delivered at the flow rate of 300 nL/min: 2–50% of solvent B in 60 min; 50–90% B in 7 min; 90% B during 10 min, and back to 2% B in 5 min. Solvent A was 100:0 H2O/acetonitrile (v/v) with 0.1% formic acid and solvent B was 0:100 H2O/acetonitrile (v/v) with 0.1% formic acid.

Peptides were infused into the mass spectrometer via dynamic nanospray probe (ThermoElectron Corp.) with a stainless steel emitter (Thermo). Typical spray voltage was 1.8 kV with no sheath and auxiliary gas flow; ion transfer tube temperature was 275°C. Mass spectrometer was operated in data-dependent mode. DDA cycle consisted of the survey scan within m/z 300–1650 at the Orbitrap analyzer with target mass resolution of 70,000 (FWHM, full width at half maximum at m/z 200) followed by MS/MS fragmentations of the top10 precursor ions. Singly charged ions were excluded from MS/MS experiments and m/z of fragmented precursor ions were dynamically excluded for further 20 s.

The software PEAKS Studio (version 7) was applied to the spectra generated by the Q-exactive plus mass spectrometer to search against either the protein sequence database UniProtKB/Trembl of the UniProt Knowledgebase (UniProtKB), limited to protein sequences of Bos Taurus. Searching for the fixed modification carbamidomethylation of cysteine and the variable post translational modifications oxidation of methionine was done with a maximum of 5 posttranslational modifications per peptide at a parent mass error tolerance of 10 ppm and a fragment mass tolerance of 0.02 Da. False discovery rate was set at 0.1%.

**Table S2. The most abundant proteins in the FND aggregation.** Sample 1 = FBS, sample 2 = 10% FBS + FNDs, sample 3 = 10% FBS + FNDs washed, sample 4 = DMEM + FBS + FNDs, sample 5 = DMEM + FBS + FNDs washed. Proteins which are marked with greenTable 1 show a molecular function related to binding to negative compounds (e.g. heparin or ATP). See next page for detailed information.

|  |  |  |  | **Sample 1** | **Sample 2** | **Sample 3** | **Sample 4** | **Sample 5** |  |
| --- | --- | --- | --- | --- | --- | --- | --- | --- | --- |
| **Protein** | **Accession** | **Avg. Mass [Da]** | **IEP** | **NpSpC_k_ [%]** | **NpSpC_k_ [%]** | **NpSpC_k_ [%]** | **NpSpC_k_ [%]** | **NpSpC_k_ [%]** | **Molecular function** |
| Hemoglobin fetal subunit beta | P02081\|HBBF_BOVIN | 15859 | 6.51 | 2.87 |  | 8.68 | 10.97 | 6.20 | Heme binding, iron binding, oxygen binding, oxygen transporter activity |
| Hemoglobin subunit alpha | P01966\|HBA_BOVIN | 15184 | 8.19 | 5.24 | 4.79 | 5.67 | 10.74 | 4.71 | Heme binding, iron binding, oxygen binding, oxygen transporter activity |
| Serum albumin | P02769\|ALBU_BOVIN | 69294 | 5.60 | 32.32 | 22.56 | 16.64 | 9.34 | 2.10 | DNA binding, drug binding, fatty acid binding, metal ion binding, oxygen binding, pyridoxal phosphate binding, toxic substance binding |
| Alpha-2-HS-glycoprotein | P12763\|FETUA_BOVIN | 38419 | 5.10 | 34.02 | 38.32 | 15.46 | 8.92 | 1.34 | Cysteine-type endopeptidase inhibitor activity |
| Apolipoprotein A-II | P81644\|APOA2_BOVIN | 11202 | 7.80 | 2.03 |  | 1.54 | 4.85 | 1.90 | Cholesterol binding, cholesterol transporter activity, high-density lipoprotein particle binding, lipase inhibitor activity, phosphatidylcholine binding, phosphatidylcholine-sterol O-acyltransferase activator activity, protein heterodimerization, triglyceride binding |
| Fibrinogen alpha chain | P02672\|FIBA_BOVIN | 67012 | 6.73 |  |  | 0.90 | 3.57 |  | N/A |
| Actin cytoplasmic 1 | P60712\|ACTB_BOVIN | 41737 | 5.29 | 0.54 |  | 0.62 | 3.13 | 1.77 | ATP binding |
| Actin cytoplasmic 2 | P63258\|ACTG_BOVIN | 41793 | 5.31 | 0.54 |  | 0.62 | 3.12 | 1.76 | ATP binding, structural constituent of cytoskeleton |
| Apolipoprotein A-I | P15497\|APOA1_BOVIN | 30276 | 5.71 |  |  | 1.14 | 3.05 | 5.02 | Beta-amyloid binding, chemorepellent activity, cholesterol binding, cholesterol transporter activity, high-density lipoprotein particle binding, high-density lipoprotein particle receptor binding, phosphatidylcholine binding, phosphatidylcholine-sterol O-acyltransferase activator activity |
| Apolipoprotein E | Q03247\|APOE_BOVIN | 35980 | 5.55 |  |  | 0.72 | 2.42 | 2.58 | Antioxidant activity, beta-amyloid binding , cholesterol binding, cholesterol transporter activity, heparin binding, lipoprotein particle binding, low-density lipoprotein particle receptor binding, metal chelating activity, phosphatidylcholine-sterol O-acyltransferase activator activity, phospholipid binding, very-low-density lipoprotein particle receptor binding |
| Cytochrome c | P62894\|CYC_BOVIN | 11704 | 9.52 | 3.88 | 10.87 | 4.41 | 2.32 | 0.29 | Electron carrier activity Source, heme binding, iron ion binding |
| Vitamin D-binding protein | Q3MHN5\|VTDB_BOVIN | 53342 | 5.36 |  |  | 2.10 | 2.14 |  | Vitamin D binding, vitamin transporter activity |
| Regakine-1 | P82943\|REG1_BOVIN | 10281 | 8.80 |  | 5.30 |  | 2.12 | 0.43 | Heparin binding |
| Insulin-like growth factor-binding protein 2 | P13384\|IBP2_BOVIN | 34015 | 7.13 |  |  |  | 2.08 | 1.51 | Insulin-like growth factor I binding, insulin-like growth factor II binding |
| Uncharacterized protein | tr\|F1N4M7\|F1N4M7_BOVIN | 68905 | 8.07 |  |  | 0.62 | 2.05 | 1.35 | Scavenger receptor activity, serine-type endopeptidase activity |
| Prothrombin* | P00735\|THRB_BOVIN | 70506 | 5.97 | 0.48 |  |  | 2.00 | 1.47 | Calcium ion binding, fibrinogen binding, serine-type endopeptidase activity, thrombospondin receptor activity |
| Gelsolin* | tr\|F1N1I6\|F1N1I6_BOVIN | 85687 | 5.86 |  |  | 0.70 | 1.59 | 2.33 | Calcium ion binding |
| Beta-2-glycoprotein 1 / Apolipoprotein H | P17690\|APOH_BOVIN | 38252 | 8.53 |  | 1.90 | 2.25 | 1.56 | 0.88 | Heparin binding Source, lipoprotein lipase activator activity, phospholipid binding |
| Inter-alpha-trypsin inhibitor heavy chain | Q3T052\|ITIH4_BOVIN | 101513 | 6.22 | 0.22 |  | 0.59 | 1.55 | 1.68 | Serine-type endopeptidase inhibitor activity |
| Platelet factor 4* | tr\|F1MD83\|F1MD83_BOVIN | 12567 | 9.30 | 3.62 | 5.78 |  | 1.30 | 1.42 | CXCR3 chemokine receptor binding, heparin binding |
| Complement factor H | Q28085\|CFAH_BOVIN | 140374 | 6.43 |  |  | 0.43 | 1.24 | 1.15 | N/A |
| Fibulin-1 * | tr\|F1MYN5\|F1MYN5_BOVIN | 77486 | 4.94 | 1.47 | 0.70 |  | 1.19 | 0.37 | Calcium ion binding, peptidase activator activity |
| Alpha-2-macroglobulin | Q7SIH1\|A2MG_BOVIN | 167575 | 5.71 |  |  | 1.95 | 1.14 | 1.01 | Serine-type endopeptidase inhibitor activity |
| Insulin-like growth factor-binding protein 6 | Q05718\|IBP6_BOVIN | 24967 | 8.73 |  |  |  | 1.09 | 0.63 | N/A |
| Fetuin-B | Q58D62\|FETUB_BOVIN | 42663 | 5.59 | 0.80 |  | 3.63 | 1.02 | 0.76 | Cysteine-type endopeptidase inhibitor activity, metalloendopeptidase inhibitor activity |
| Apolipoprotein C-III | P19035\|APOC3_BOVIN | 10692 | 5.02 |  |  |  | 1.02 | 0.84 | Lipase inhibitor activity, phospholipid binding |
| Uncharacterized protein | tr\|Q3ZBS7\|Q3ZBS7_BOVIN | 53575 | 5.92 |  |  |  | 0.91 | 1.06 | Extracellular matrix binding, polysaccharide binding, scavenger receptor activity |
| Protein AMBP | P00978\|AMBP_BOVIN | 39235 | 7.81 | 2.03 | 2.78 |  | 0.83 |  | Heme binding, IgA binding, protein homodimerization activity, serine-type endopeptidase inhibitor activity, small molecule binding |
| Alpha-fetoprotein | Q3SZ57\|FETA_BOVIN | 68588 | 5.92 |  |  | 2.01 | 0.79 | 0.28 | Metal ion binding |
| Tetranectin* | Q2KIS7\|TETN_BOVIN | 22144 | 5.47 |  |  |  | 0.74 | 1.06 | Calcium ion binding, carbohydrate binding, heparin binding |

*Ca-binding function

In table S2 an overview of the identified proteins given, ordered by the 30 most abundant proteins of sample 4 (DMEM + FBS+ FNDs). The full list of proteins of samples is available upon request. A semi-quantitative assessment of (relative) protein amounts was conducted using normalized spectral counts given by the following equation: ^1–3^

$$NpSpC_{k}=\left( \frac{{(SpC/M_{W})}_{k}}{\sum_{i=1}^{n} {(SpC/M_{W})}_{i}} \right)*100$$

where NpSpC_k_ is the normalized percentage of spectral count for protein k, SpC is the spectral count identified, and M_W_ is the molecular weight (in Da) of the protein k. The protein corona does not reflect the relative abundance of proteins of sample 1, which is the pure FBS, suggesting some specificity of the adsorption process.

Sample 1 revealed the most abundant proteins (also known from literature^2,4^): Serum albumin and Alpha-2-HS-glycoprotein make up 66% of the normalized spectral counts. Sample 2 and Sample 3 reflect that with pure FBS, hardly any aggregation is happening. The protein pattern closely resembles the one from sample 1.

Amongst the proteins of sample 4, Prothrombin, Gelsolin, Platelet factor 4, Fibulin-1 and Tetranectin (marked with a star) are known to be binding to calcium, which is present in DMEM medium and was proven to play a role in the agglomeration process by XPS analysis.

Identifying hard corona:

In order to differentiate between hard and soft corona we also performed sedimentation through a sucrose cushion to remove loosely bound proteins. To this end we first mixed 5 ul of 20mg/ml FND25 with 495 ul of DMEM Complete Medium. Then we incubated the samples for 5 minutes and centrifuged them through a 0.7M Sucrose cushion (500 ul) for 20 minutes at 15.400xG. After removing the supernatant we resuspended the pellet in 1x PBS (pH 7.4) and centrifuged it again 20 min at 15.400xG. This step was repeated once. After the last washing step, the samples were stored at -20 degrees until freeze drying (for 2 hours). For freeze drying: first the samples were cooled down to -50 degrees for 1 hour and 15 minutes. Then a vacuum of 0.055 mbar was applied and the samples were left over night. The next day the samples were gradually brought to room temperature and the vacuum was released. Then we continued the protein analysis as described for the other samples in the main manuscript.


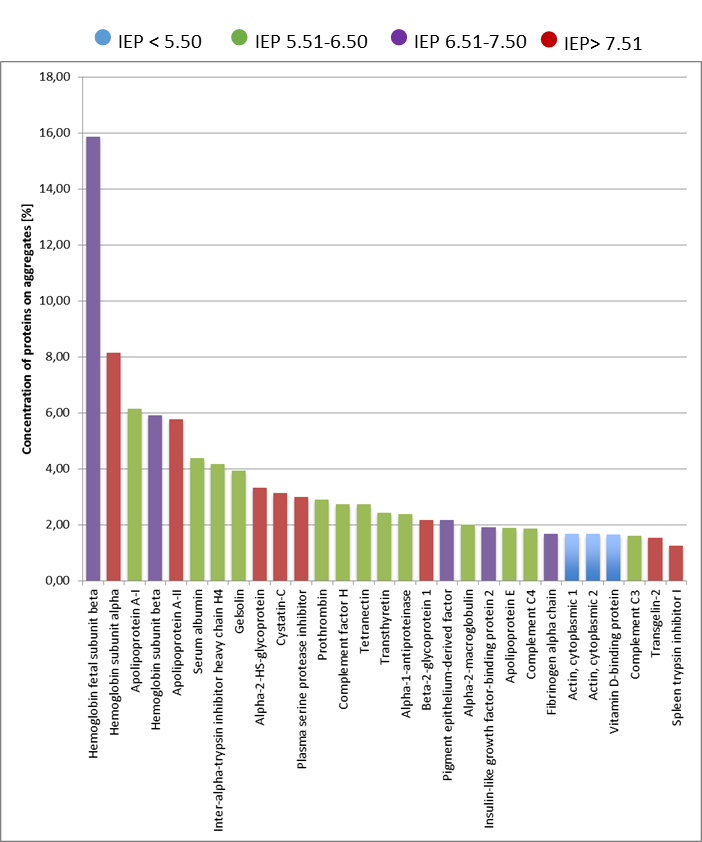


**Figure S3**. Hardest bound proteins identified by sedimenting diamond aggregates through a sucrose cushion

**XPS Methods**

Instruments, Scienta Scientific, Uppsala, Sweden), X-ray production set to 10 kV, 22 mA with a spot size of 250 by 1000 µm using an aluminum anode. Wide scans were performed with an energy range of 0 to 1200 eV at low resolution (pass energy, 150 eV). The area under each peak, after Shirley background subtraction, was used to calculate peak intensities, yielding elemental surface concentration ratios for nitrogen (N), oxygen (O), and phosphorus (P) to carbon (C). Narrow scans for C, O and N were made at a pass energy of 50 eV, these where used for peak fitting of the carbon and oxygen peak. The sample was checked for contamination by monitoring the increase of the C-C peak after repeating measurements of the carbon peak.


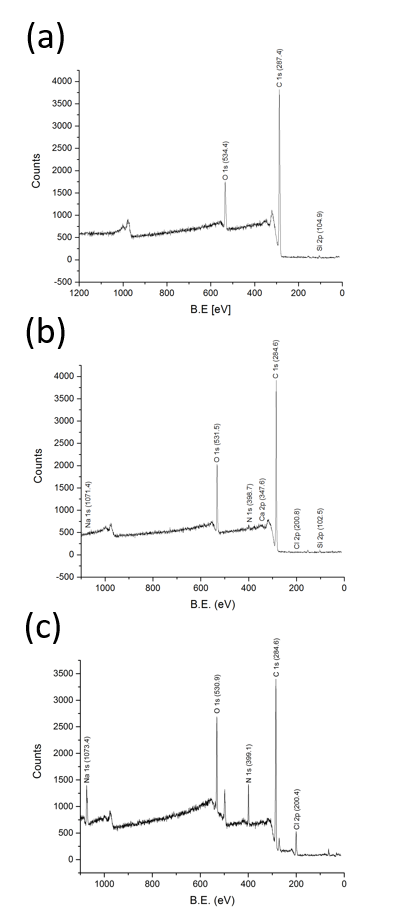


**Figure S3**. **XPS spectra of the aggregates**. (a) Pure FND: Spectrum of an oxygen-terminated acid-cleaned FND dominated by carbon and oxygen (with traces of N, P and Si). (b) FND in DMEM. Sodium chloride identified as the main component responsible for the salting out effect. Small amounts of N, Ca and Si present. (c) FND in DMEM + 10% FBS. Also here, sodium chloride remains the main compound in the aggregates (apart from the carbon mainly present as diamond, protein and amino acids).

We can see the typical XPS spectrum of oxygen-terminated and acid-cleaned FNDs. Small amounts of P, N and Si (contaminations, possibly also from the production process of the FNDs) The peak fitting of carbon and oxygen reveal the presence of carboxylate groups and carboxylic acid and alcohol groups, all of which are likely to be present on the surface. Figure 5b on the other hand shows the presence of inorganic salts, especially sodium chloride alongside calcium, nitrogen and again small amounts of Si. As seen in Figure 5c, with FBS present, also sodium chloride alongside N are the main inorganic elements to be found. In such a complex mixture as DMEM (+ 10% FBS) peak fittings of carbon and oxygen only reveal the adsorption of proteins and other organic components present in the medium: Further differentiation is virtually impossible. Nitrogen is present both in form of inorganic nitrogen salts as well as amino acids and proteins (in case of Sample (c)).

**Table S3.** Elements present at the surface of FND/medium aggregates.

| present elements | FNDs (control sample) | aggregates DMEM without FBS | aggregates DMEM with serum proteins |
| --- | --- | --- | --- |
| Carbon | 86.07% | 80.39% | 65.73% |
| Oxygen | 11.65% | 12.59% | 16.45% |
| Sodium |  | 2.31% | 4.82% |
| Nitrogen | 0.45% | 1.93% | 8.39% |
| Chloride |  | 1.31% | 4.61% |
| Calcium |  | 0.64% |  |
| Silicon | 1.65% | 0.83% |  |
| Phosphrous | 0.18% |  |  |


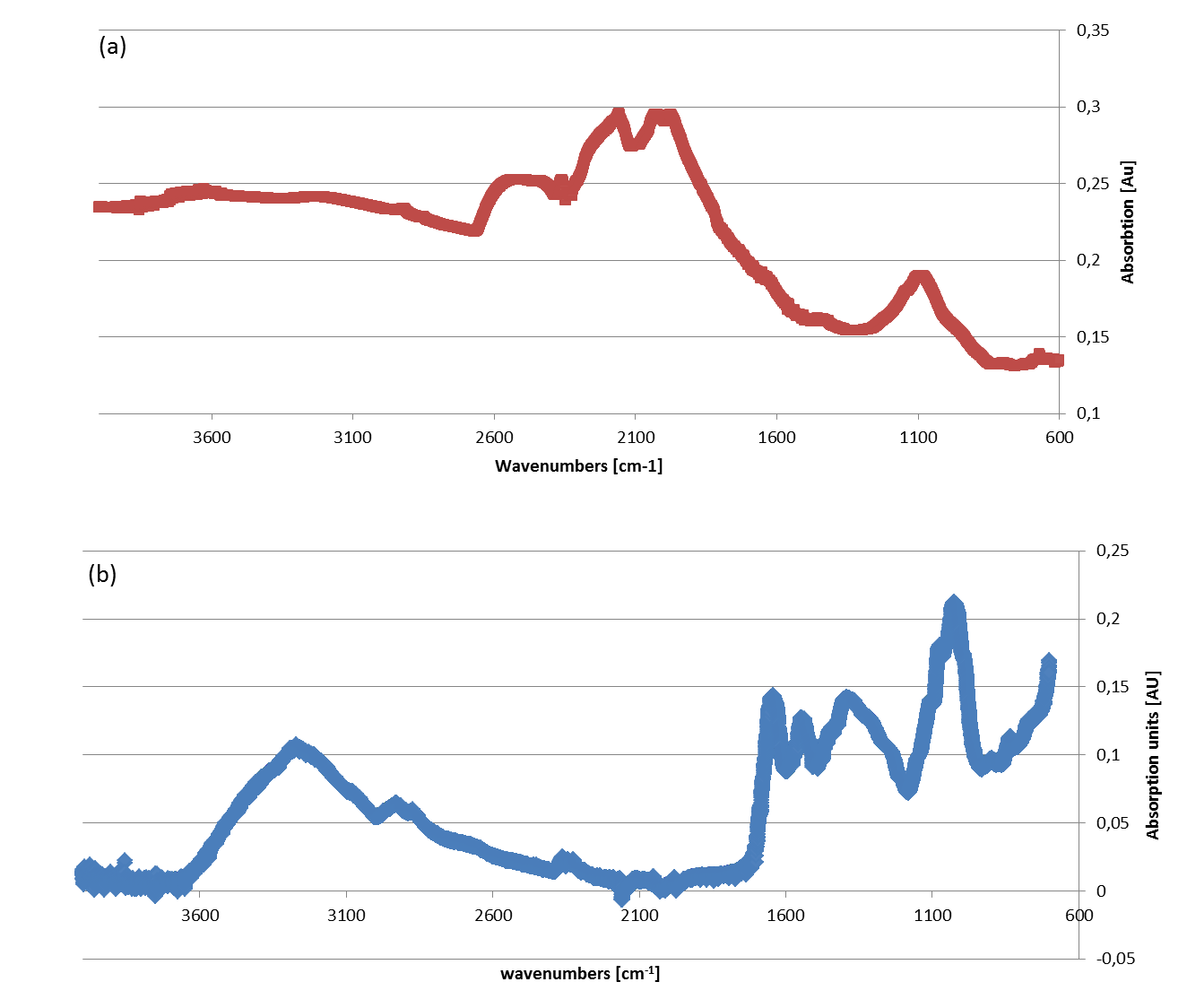


**Figure S4.** IR spectra of (a) pure diamond powder and (b) aggregates formed in complete DMEM +FBS. The second spectrum is dominated by protein bands.

**REFERENCES**

(1) Sakulkhu, U.; Maurizi, L.; Mahmoudi, M.; Motazacker, M. M.; Vries, M.; Gramoun, A.; Beuzelin Ollivier, M.-G.; Vallee, J.-P.; Rezaee, F.; Hofmann, H. *Nanoscale* **2014**, No. June.

(2) Usawadee, S.; Morteza, M.; MauriziLionel; Geraldine, C.; Margarethe, H.-A.; Marcel, V.; Mahdi, M.; Farhad, R.; Heinrich, H. *Biomater. Sci.* **2015**, *3* (2), 265–278.

(3) Zhu, W.; Smith, J. W.; Huang, C. M. *J. Biomed. Biotechnol.* **2010**, *2010*.

(4) Zheng, X.; Baker, H.; Hancock, W. S.; Fawaz, F.; McCaman, M.; Pungor, E. *Biotechnol. Prog.* **2006**, *22* (5), 1294–1300.
